# Tuberculosis outbreak in a pre-primary and primary school in Barcelona, Spain

**DOI:** 10.3389/fpubh.2026.1789425

**Published:** 2026-04-01

**Authors:** Raquel Prieto-García, Joan Pau Millet, Claudia Broto, Elisa Lara, María Espiau, Antoni Soriano-Arandes, Maria-Luiza de Souza-Galvão, Ma Angeles Jiménez-Fuentes, Cristina Domingo, Rosa Mercè Vileu Vallverdú, Verónica Saludes, Antoni E. Bordoy, Poppy J. Hesketh-Best, David Panisello Yagüe, Elisa Martró, Laura Gavaldà Mestre, Elisabet Sicart Torres, Helena Martínez Alguacil, Mar López Espinilla, Pere-Joan Cardona, Pere Simón, Cristina Rius

**Affiliations:** 1 Department of Experimental Sciences i de la Salut (DCEXS), Universitat Pompeu Fabra, Barcelona, Spain; 2 Servei d'Epidemiologia (SEPID), Agència de Salut Pública de Barcelona (ASPB), Barcelona, Spain; 3 Institut de Recerca Sant Pau (IIB Sant Pau), Barcelona, Spain; 4 Biomedical Research Consortium in Epidemiology and Public Health Network (CIBERESP), Madrid, Spain; 5 Unitat de Tuberculosi Drassanes-Vall Hebron, Centre de Salut Internacional i Malalties Transmissibles Drassanes—Vall d'Hebron, Institut Català de la Salut, Barcelona, Spain; 6 Unitat de Patologia Infectosa i Immunodeficiències de Pediatria, Hospital Universitari Vall d'Hebron, Barcelona, Spain; 7 Servei de Vigilància Epidemiològica i Resposta a Emergències de Salut Pública al Camp de Tarragona i Terres de l'Ebre, Tarragona, Spain; 8 Servei de Microbiologia, Laboratori Clínic Metropolitana Nord, Institut de Recerca i Hospital Germans Trias i Pujol (IGTP), Barcelona, Spain; 9 Department of Genetics and Microbiology, Autonomous University of Barcelona, Barcelona, Spain; 10 Servei de Prevenció i Control de la Tuberculosi i Programes Específics, Subdirecció General de Vigilància i Resposta a Emergències de Salut Pública, Secretary of Public Health, Department of Salut, Generalitat of Catalonia, Barcelona, Spain; 11 CIBER in Respiratory Diseases (CIBERES), Carlos III Institute of Health, Madrid, Spain

**Keywords:** child, contact tracing, disease outbreaks, pulmonary, schools, tuberculosis, whole genome sequencing

## Abstract

**Background:**

In June 2022, a case of non-bacillary pulmonary tuberculosis (TB) was reported in a 10-year-old girl attending a primary school in Barcelona. A second pediatric case from the same school was identified in October 2022, prompting the declaration of a TB outbreak and the initiation of an epidemiological investigation.

**Methods:**

Contact tracing was conducted using the concentric circles approach, targeting household contacts, classmates, and school staff. Tuberculin skin tests were administered and read 72 h later. Whole genome sequencing of Mycobacterium tuberculosis isolates was performed to identify potential source cases and transmission chains.

**Results:**

Among 218 individuals screened, 30 TB infections and 4 TB disease cases were diagnosed. The highest prevalence occurred in grades EP4 and EP5 (ages 9–10), with a significantly higher risk compared to other grades. No adult cases linked to the school were identified. Later, a genomic cluster of 15 cases was detected across Catalonia, suggesting a super-spreading event. However, no direct epidemiological link to the school was established.

**Conclusions:**

This outbreak highlights the challenges of TB detection in pediatric populations and the importance of genomic surveillance in tracing transmission. Despite extensive investigation, the source remains unidentified, underscoring the complexity of TB dynamics in urban settings.

## Introduction

Tuberculosis (TB) is an airborne disease caused by Mycobacterium tuberculosis (MTB). Transmission depends on community prevalence, frequency and proximity of contact, host factors (e.g., diabetes, HIV, and age), and environmental determinants such as overcrowding and poor ventilation (1, 2).

In 2023, TB affected 10.8 million people globally, including 1.2 million children under 15 years (11.5%) (3). In 2022, pediatric TB incidence was 3.9 per 100,000 in the EU (4) and 5.8 per 100,000 in Spain (5). In Barcelona, 10 cases were reported in children under 15 in 2023, compared with 20 in 2022 and 8 in 2021 (6, 7).

The burden of TB in childhood underscores the need for effective prevention and early diagnosis to avoid severe forms such as tuberculous meningitis or miliary TB (8, 9). Contact tracing based on concentric circles should explicitly consider environmental conditions (ventilation, crowding) and duration of exposure, which influence transmission risk (10).

Integrating whole-genome sequencing (WGS) with contact tracing refines transmission mapping, can suggest directionality, and helps identify unsampled cases, thereby informing control strategies and flagging potential superspreading (11, 12). However, contact tracing in schools may be limited if the index case has low bacillary load or exposures occur in shared spaces with many contacts (11, 13, 14).

This study analyses a TB outbreak in a pre-primary and primary school in Barcelona (2022), characterizes transmission, and highlights the value of combining traditional contact tracing with genomic epidemiology to understand circulating strains in Catalonia.

## Detection of the outbreak

In June 2022, a 10-year-old EP5 student in Barcelona was reported with paucibacillary pulmonary TB, with clinical and radiological signs but no microbiological confirmation; her TST measured 20 mm with vesiculation.

**Figure 1:**
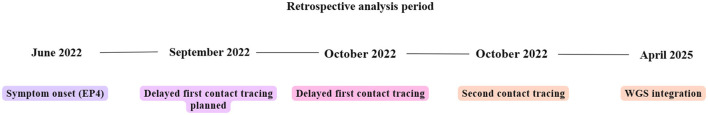
Timeline of key epidemiological and laboratory events in the tuberculosis outbreak.

In October, a second case occurred: a 9-year-old EP4 student, symptomatic since May (fever, cough), diagnosed 10 days after onset and without prior school contact tracing. Her chest X-ray showed non-cavitated lesions, and sputum culture was MTB-positive (Figure 1: Timeline).

Following these two cases, an outbreak was declared and an epidemiological investigation was initiated to identify the source case.

## Methods

### Definitions

Primary case: first TB case identified in an outbreak; may not be the actual transmitter.

Index case: case that triggers contact tracing; not necessarily the source.

Source case: individual proven to transmit MTB based on epidemiological or molecular evidence.

Positive TST and follow-up: TST ≥5 mm was considered positive. Participants were referred to UTBD (Unit of Tuberculosis Drassanes) for chest X-ray and IGRA (if BCG-vaccinated) to confirm TBI or rule out false positives. Following national/WHO-endorsed criteria, a ≥5 mm cut-off was applied regardless of BCG history; among BCG-vaccinated contacts with positive TST, IGRA was required to confirm TBI. IGRA did not modify TB disease classification, which relied on clinical and microbiological criteria (5).

### Study design

Mixed design combining: (1) retrospective outbreak investigation (May 2022–April 2025); (2) two cross-sectional contact-tracing rounds in October 2022; and (3) genomic epidemiology through WGS of culture-positive isolates.

All components were integrated to characterize transmission dynamics and identify potential source and secondary cases.

## Traditional epidemiological investigation and data collection

Contact tracing followed the concentric-circle approach covering household and school contacts (classmates, teachers, and staff). The ASPB (Agència de Salut Pública de Barcelona) Epidemiology Service conducted the first investigation in early October 2022, performing TST and readings at 72 h.

Expansion beyond the first circle was triggered when ≥10% TST positivity or at least one secondary TB case was identified, and when shared indoor exposure in poorly ventilated environments suggested elevated transmission risk. Both criteria were met in the first investigation (40% TST positivity and one TB case in EP5), so the second round included all students, staff, and former pupils potentially exposed during the 2021–2022 academic year (15).

Both investigations occurred beyond the ideal window period due to logistical issues. The first revision should have taken place at the end of June 2022, but the school year had ended; it was therefore performed in September 2022. Notification of a possible source case in May 2022 did not reach the tracing team until after the first review. Adults linked to the school and family members were assessed, and TB cases from the neighborhood and Tarragona were reviewed.

**Figure 2:**
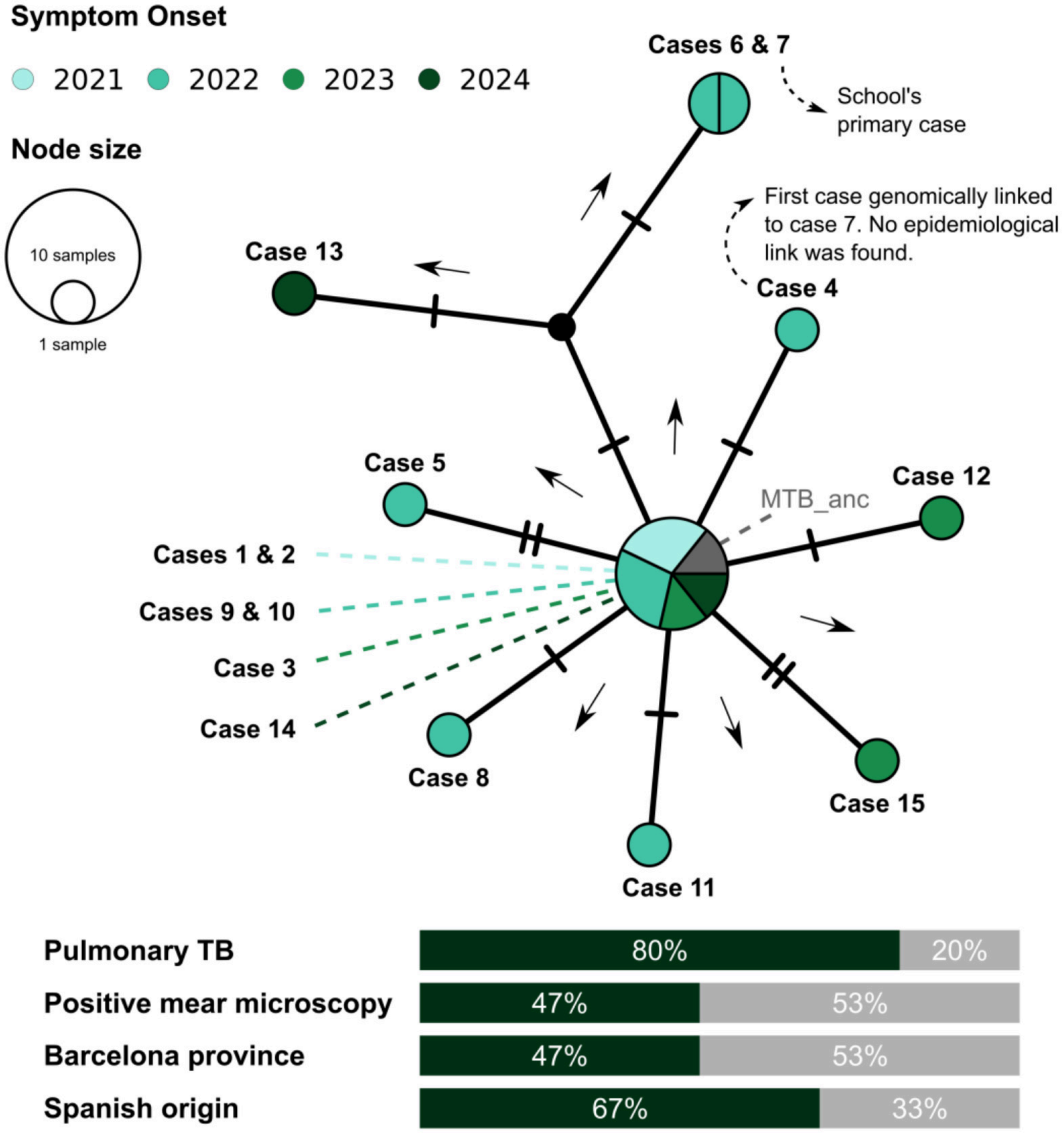
Median-joining network of MTB strains in the genomic cluster identified by WGS ( ≤ 5 SNPs). It includes all cases up to April 2025. Circles represent nodes of identical isolates; size reflects case count and is split by year of symptom onset. Hash lines indicate SNP distance. Arrows indicate inferred transmission direction. Small black nodes denote inferred unobserved cases. Selected clinical and demographic variables are summarized at the bottom. ^*^First case identified in the school; MTB_Anc: reconstructed ancestor of the genomic cluster; Mixed TB, pulmonary and extrapulmonary involvement; CXR, chest X-ray (normal, abnormal non-caitatd, cavitated); BK, bacilloscopy for MTB; SO, symptom onset (month/year); AR, Tarragona; BCN, Barcelona.

WGS of the 9-year-old's isolate was requested from the Microbiology Service of Hospital Germans Trias i Pujol (HUGTiP), and UTBD studied family contacts (Figure 2).

## Data analysis

We conducted a cross-sectional analysis to estimate TB and TBI prevalence by grade. Excess risk in EP4–EP5 was assessed with odds ratios (OR) and 95% confidence intervals; significance was tested with chi-square (*p* < 0.05). Analyses were performed in RStudio (v1.3.1093).

Individuals absent or declining TST (*n* = 41) were recorded as non-tested and excluded from prevalence estimates but retained for participation rates. Analytical comparisons included only valid TST results. Non-tested individuals were distributed across grades without clustering in EP4–EP5, suggesting limited bias in the observed excess risk.

## Genomic epidemiology

HUGTiP performed WGS on the MTB-positive sputum culture from the 9-year-old (the only culture-positive school case). The sequence was compared within the TB-SEQ strategy for genomic epidemiology in Catalonia, launched in late 2022 (16). Lineage and resistance were analyzed (e.g., TB-Profiler v6.6.3) (17), and phylogenies inferred with IQ-TREE2 (GTR+G4, 1,000 bootstraps) (18, 19). Clusters were defined as ≥2 cases within 0–5 SNPs.

## Environmental research

Inspection of the building (classrooms and shared spaces, including the dining hall) revealed poor ventilation and smaller-than-expected classrooms.

## Ethical considerations

Demographic and clinical data were collected using the ASPB epidemiological questionnaire and analyzed anonymously. Statistical analysis was performed retrospectively (March–September 2023). The team had no access to identifiable information. Written consent was obtained from families of minors; adults gave verbal consent.

The study was approved by the Parc de Salut Mar Ethics Committee (CEIm-PSMAR, 2023/11081) and complied with the Helsinki Declaration (20); Organic Law 7/2021 (21), and Directive (EU) 2016/680 (22).

## Results

The first round (early October 2022) targeted the EP5 class of the index case: TST was performed in 24 children and 6 teachers; 12 (40%) were positive (Table 1).

**Table 1:** Screening participation and TST positivity by round and grade.

Round	Grade	TST programmed	TST carried out	TST + women *n* (%)	TST + men *n* (%)	TST + total *n* (%)	Total per grade (*n*)
First	EP5	25	24	6 (25.0)	4 (16.7)	10 (41.7)	30
First	Teachers EP5	6	6	2 (33.3)	0 (0.0)	2 (33.3)	6
Second	PP4	16	11	0 (0.0)	0 (0.0)	0 (0.0)	11
Second	PP5	24	19	1 (5.3)	0 (0.0)	1 (5.3)	19
Second	EP1	26	22	2 (9.1)	3 (13.6)	5 (22.7)	22
Second	EP2	24	21	0 (0.0)	3 (14.3)	3 (14.3)	21
Second	EP3	25	24	0 (0.0)	2 (8.3)	2 (8.3)	24
Second	EP4	25	23	6 (26.1)	4 (17.4)	10 (43.5)	23
Second	EP6	26	25	0 (0.0)	0 (0.0)	0 (0.0)	25
Second	Center staff	26	15	2 (13.3)	0 (0.0)	2 (13.3)	15
Second	Alumni (ESO)	26	17	1 (5.9)	1 (5.9)	2 (11.8)	17
Second	All grades/staff (round total)	218	177	12	13	25 (14.1)	177

1. Non-tested (absent/declined) are excluded from prevalence estimates and shown in denominators only when reporting participation rates.

2. Where expected counts <5, p-values are from Fisher's exact test.

3. Denominators are harmonized across tables; discrepancies reflect exclusions specified above.

PP, pre-primary education; EP, primary education; ESO, compulsory secondary education; TST, tuberculin skin test.

The second round (mid-October) screened the entire school (218 individuals: students, staff, former pupils). Six new students were excluded; 16 did not undergo TST. We observed 25 TST-positive individuals (14.1%) among students and staff (Table 1).

Overall, 30 TBI cases (14.4%) and four TB cases were diagnosed (Table 2). In EP5 (index-class), nine TBI (30%) and one TB case (3.3%) occurred; in EP4, seven TBI (30.4%) and three TB cases (13%). TBI distribution in EP4–EP5 differed significantly from other grades (OR 4.3; 95% CI 2.2–10.6; *p* < 0.001).

**Table 2:** TBI and TB cases by round and grade.

Round	Grade	TST carried out	TBI women *n* (%)	TBI men *n* (%)	TBI total *n* (%)	TB women *n* (%)	TB men *n* (%)	TB total *n* (%)	Total per grade (*n*)
First	EP5	24	5 (20.8)	3 (12.5)	8 (33.3)	1 (4.2)	0 (0.0)	1 (4.2)	24
First	Teachers EP5	6	1 (16.7)	0 (0.0)	1 (16.7)	0 (0.0)	0 (0.0)	0 (0.0)	6
Second	PP4	11	0 (0.0)	0 (0.0)	0 (0.0)	0 (0.0)	0 (0.0)	0 (0.0)	11
Second	PP5	19	1 (5.3)	0 (0.0)	1 (5.3)	0 (0.0)	0 (0.0)	0 (0.0)	19
Second	EP1	22	2 (9.1)	3 (13.6)	5 (22.7)	0 (0.0)	0 (0.0)	0 (0.0)	22
Second	EP2	21	0 (0.0)	2 (9.5)	2 (9.5)	0 (0.0)	0 (0.0)	0 (0.0)	21
Second	EP3	24	0 (0.0)	2 (8.3)	2 (8.3)	0 (0.0)	0 (0.0)	0 (0.0)	24
Second	EP4	23	3 (13.0)	4 (17.4)	7 (30.4)	3 (13.0)	0 (0.0)	3 (13.0)	23
Second	EP6	25	0 (0.0)	0 (0.0)	0 (0.0)	0 (0.0)	0 (0.0)	0 (0.0)	25
Second	Center staff	15	2 (13.3)	0 (0.0)	2 (13.3)	0 (0.0)	0 (0.0)	0 (0.0)	15
Second	Alumni (ESO)	17	1 (5.9)	1 (5.9)	2 (11.8)	0 (0.0)	0 (0.0)	0 (0.0)	17
Second	All grades/staff (round total)	177	9 (5.1)	12 (6.8)	21 (11.9)	3 (1.7)	0 (0.0)	3 (1.7)	177
Total	All rounds combined	207	15 (7.2)	15 (7.2)	30 (14.4)	4 (1.9)	0 (0.0)	4 (1.9)	207

1. Non-tested (absent/declined) are excluded from prevalence estimates and shown in denominators only when reporting participation rates.

2. Where expected counts <5, p-values are from Fisher's exact test.

3. Denominators are harmonized across tables; discrepancies reflect exclusions specified above.

PP, pre-primary education; EP, primary education; ESO, compulsory secondary education; TST, tuberculin skin test.

**Table 3:** Odds ratio of TBI according to the relationship with the center (EP5 and EP4 vs. other courses).

Relationship with the center	n	TBI	%	OR	IC_lower	IC_upper	*p*-value
Other courses	154	14	9.1				
EP5 and EP4	53	16	30.2	4.3	2.2	10.6	<0.001

No TB cases were found among adults, family members, or staff linked to the school in 2021–2022.

WGS classified the 9-year-old's strain as lineage 4.10 (Case 7). TB-SEQ identified an adult case in Tarragona (November 2022) separated by 3 SNPs from the pediatric case, but no epidemiological link was confirmed. By April 2025, 15 cases formed a genomic cluster (Barcelona: 7; Tarragona: 8), including 10 autochthonous and 5 from Morocco, Equatorial Guinea, Peru, and Romania. A median-joining network showed a star-like topology suggestive of a superspreading context, though no direct link to the school was demonstrated (Figure 2).

## Discussion

We describe a school TB outbreak analyzed through classical epidemiology (two contact-tracing rounds) and population genomic epidemiology. Despite extensive investigation, the source case could not be confirmed.

TB in children indicates recent transmission (6). Given this and the suspected characteristics of a potential source, we hypothesized an adult with some link—continuous or sporadic—to the school. Minors rarely transmit MTB and, when they do, they usually show adult-like features. No such adult case was identified among school-linked contacts.

We considered whether the 9-year-old culture-positive case could have been the source. However, no cohabiting family member was infected, and she was diagnosed only 10 days after symptom onset and was not attending school at that time.

Transmission in schools is well documented, particularly in younger children (23–29). This outbreak occurred in a small center with poor ventilation. TB disease and TBI concentrations were highest in the two classrooms of the index and EP4 cases, whose students also shared a confined dining area, increasing cumulative exposure. These findings align with systematic reviews reporting higher TBI prevalence among classroom contacts of the index case and in poorly ventilated indoor settings (13, 30).

WGS linked the pediatric strain to a broader regional cluster including an adult case with a closely related strain. No epidemiological link was found, so the origin of the school outbreak remains unclear. The star-like cluster topology suggests a superspreading event may have preceded the school cases, but a direct connection was not demonstrated. Notably, the transmission chain containing the schoolgirl did not accumulate new cases over 3 years, suggesting that two rounds of contact tracing and appropriate treatment prevented further transmission.

Strengths include a comprehensive approach combining classical and molecular epidemiology, inclusion of pediatric and adult cases, and explicit consideration of environmental factors. Genomic integration provided context by linking the school outbreak to a broader cluster, despite the absence of a confirmed source.

Limitations include the timing of both investigations outside the optimal window period, a lack of additional culture-positive cases for sequencing, and some refusals. Screenings months after exposure may overestimate TBI prevalence (due to infections acquired later yet still detected in October 2022) and underestimate recent transmission (due to window-period false negatives). Changes in class composition and potential recall bias may have hindered the identification of exposures. Missingness was likely non-differential across grades, but some misclassification cannot be excluded. Although IGRA mitigated false-positive TST among BCG-vaccinated students, the absence of IGRA for all participants may have introduced variability. Precision was limited in some estimates (e.g., wide CIs for the EP4–EP5 association), and residual confounding cannot be ruled out.

Rapid census and contact tracing are essential in educational settings with close contact. Systematic assessment of shared spaces and ventilation should be prioritized. Based on preliminary findings, expansion beyond the initial concentric circle may be warranted (8, 9).

Investigations should be conducted whenever the index case has pulmonary TB, assessing both potential source cases and secondary cases or TBI. Early case identification and scaling investigations according to observed risk can prevent further transmission.

## Collaborators

The members of TB-SEQ Working Group are as follows: Servei de Microbiologia, Laboratori Clínic Metropolitana Nord, Institut de Recerca i Hospital Germans Trias i Pujol (IGTP), Barcelona: L. Soler, A. Antuori, A. Paris, S. González-Gómez, G. Clarà. Laboratori de Referència de Catalunya, El Prat de Llobregat, Barcelona: S. Esteban-Cucó and E. Vicente. Hospital de la Santa Creu i Sant Pau, Barcelona: M. Garrigó. Hospital Universitari Vall d'Hebron, Barcelona and CIBERINFECT, Madrid: M. T. Tórtola. Universitat de Barcelona and Hospital Clínic-ISGlobal, Barcelona and CIBER en Enfermedades Infecciosas (CIBERINFEC), Madrid: G. Tudó and J. González. Hospital Universitari Joan XXIII, Tarragona: M. Galofré. Laboratorio Echevarne, Barcelona: Joan Ramon Agüera.

## Data Availability

Sequence data have been deposited in the Short Read Archive (SRA) under the BioProject accession number PRJNA1371778. In addition, epidemiological surveillance data used in the study are held in a secure environment within the Epidemiology Service of the Agència de Salut Pùblica de Barcelona. As these data originate from population-based epidemiological surveillance systems, they are not openly available. However, they can be made accessible upon reasonable request, following the agency's data protection and confidentiality protocols.
